# Editorial: Toward Sustainable Energy: The Unique Role of Rare Earth Oxides

**DOI:** 10.3389/fchem.2019.00478

**Published:** 2019-07-03

**Authors:** Cristina Artini, Paolo Mele, Nobuhito Imanaka

**Affiliations:** ^1^Department of Chemistry and Industrial Chemistry, University of Genoa, Genoa, Italy; ^2^Consiglio Nazionale delle Ricerche - Institute of Condensed Matter Chemistry and Energy Technologies (CNR-ICMATE), Genoa, Italy; ^3^Shibaura Institute of Technology (SIT) Research Laboratories, Saitama, Japan; ^4^Graduate School of Engineering, Osaka University, Suita, Japan

**Keywords:** sustainable energy, rare earth oxides, SOFC (solid oxide fuel cell), superconductivity, luminescence, catalysis, CO_2_ capture

Sustainable energy is one of the keywords of the future. The EU energy policies, just as an example, indicate as a long-term strategy the goal of net-zero greenhouse gas emissions by 2050 (European Commission, 2018). This complicated but unavoidable pathway requires an effective contribution both from basic and applied research. Besides, it is clear that greenhouse emissions concerns cannot be swept away by the development and employment of just one energy form; on the contrary, multiple technologies have to be invoked in order to face the environmental issue. Among these, the fuel cell technology and in general all the techniques aiming at CO_2_ capture and reuse, as well as high temperature superconductivity and to a certain extent even photoluminescence, play a key role in reducing the carbon footprint of our society. The removal of polluting compounds from wastewater is a further challenge, which addresses the environmental problem from a different viewpoint.

All of these items share a common ground, consisting in strongly guiding fundamental science toward the search for new and progressively more efficient materials. In addition, among materials, rare earth oxides occupy a central place in the advancement of all the aforementioned technologies, due to their manifold and peculiar properties.

Doped ceria, for instance, is widely studied as an electrolyte in solid oxides fuel cells operating in the intermediate temperature range (673–973 K). The free movement of oxygen anions through the CeO_2_-based lattice, in fact, induces high values of ionic conductivity at temperatures significantly lower than the ones observed when using Y_2_O_3_-stabilized ZrO_2_; this phenomenon in principle enables a substantial reduction of the cell thermal stress and hence is expected to lengthen the device lifetime. The choice of the most proper doping ion and of its content, has been widely studied in recent years, with a particular focus on the creation of defect clusters, which are known to reduce and even suppress ionic conductivity (Artini, 2018).

In tandem with the production of renewable energy, the capture, storage and reuse of emitted CO_2_ are the foundation of sustainable development. Within this framework, CeO_2_-based oxides, acting as active supports for catalytic metals, form a class of materials with excellent catalytic activity, mainly due to the Ce^4+^/Ce^3+^ redox properties, as well as to the presence of surface defects, which enhance the oxide reactivity through the presence of oxygen vacancies (Trovarelli and Fornasiero, 2013). In the methanation process of CO_2_, these are indeed the properties which promote and catalyze the reaction: the surface of the oxide favors the adsorption of CO_2_ by exposing Ce^3+^ ions, while the presence of vacancies accelerates the chemical transformation.

High temperature superconductivity in Cu-based complex oxides (Bednorz and Müller, 1986) has been long and thoroughly investigated by the scientific community due to its huge potential. Even if the mechanism underlying the high temperature lossless transmission of electricity in such materials has not yet completely revealed its nature, the presence of a rare earth oxide layer within the crystal structure is considered as highly desirable. The most famous high temperature superconductor, namely YBCO (YBa_2_Cu_3_O_7−x_), clearly contains yttrium oxide, while the REBCO family relies on the presence of a rare earth (RE) oxide.

The outstanding electronic properties of rare earths are also responsible for the luminescence and photoluminescence properties of many lanthanide oxides, such as perovskites (Artini, 2017). Rare earth ions, being characterized by partially filled 4*f* shells, which are highly shielded by the outer 5*s* and 5*p* orbitals, generate a large number of quantized energy levels scarcely affected by the host crystal field. If properly excited, electrons populating these orbitals can emit in a region of the electromagnetic spectrum different from the one where absorption took place. Besides, in long-lasting phosphors the absorbed energy is stored in trap-centers and the release can last many hours or even days, thus giving rise to an actual form of energy storage.

Again with reference to environmental issues, several rare earth oxide-based compounds are also active promoters of cleanup reactions. CeO_2_-ZrO_2_ solid solutions are studied as optimal catalysts for the oxidation of organic pollutants dissolved in wastewater, such as for example phenol and its derivatives. Even in this case, the Ce^4+^/Ce^3+^ redox properties are responsible for the ability of CeO_2_ to store and then release oxygen, thus providing this oxide a leading role even in this field. Similarly, REFe_2_O_4+x_-based catalysts are characterized by a remarkable ability to intercalate oxygen, which makes them interesting candidates to catalyze oxidation reactions of organic compounds.

This thematic collection provides a comprehensive overview of the state-of-the-art related to the employment of rare earth oxides in the branch of materials science addressing environmental issues and sustainable development. Topics such as fuel cell technology, CO_2_ capture and reuse, superconductivity, luminescence, and catalysis oriented toward cleanup reactions, are extensively treated.

## Author Contributions

CA wrote the first draft of the manuscript. All authors contributed to manuscript revision, read, and approved the submitted version.

### Conflict of Interest Statement

The authors declare that the research was conducted in the absence of any commercial or financial relationships that could be construed as a potential conflict of interest.
